# Supplementary material for: Problematic use of the internet, smartphones, and social media among medical students and relationship with depression: An exploratory study
Source: PLoS One. 2023 May 26;18(5):e0286424. doi: 10.1371/journal.pone.0286424 (PMC10218731; doi:10.1371/journal.pone.0286424)
Supplement: Attachment — Submitted filename: Response to Reviewers.docx [file pone.0286424.s001.docx]

**Responses to Editors’ comments**

**Comment:** Please ensure that your manuscript meets PLOS ONE's style requirements, including those for file naming. The PLOS ONE style templates can be found at

<https://journals.plos.org/plosone/s/file?id=wjVg/PLOSOne_formatting_sample_main_body.pdf> and

<https://journals.plos.org/plosone/s/file?id=ba62/PLOSOne_formatting_sample_title_authors_affiliations.pdf>

**Response**: We have edited and renamed all files as required.

**Comment**: Please provide additional details regarding participant consent. In the ethics statement in the Methods and online submission information, please ensure that you have specified what type you obtained (for instance, written or verbal, and if verbal, how it was documented and witnessed). If your study included minors, state whether you obtained consent from parents or guardians. If the need for consent was waived by the ethics committee, please include this information.

**Response**: Consent details have been added to the revised Methods section of the manuscript.

**Comment**: Your ethics statement should only appear in the Methods section of your manuscript. If your ethics statement is written in any section besides the Methods, please delete it from any other section.

**Response:** The ethics statement under ‘Declarations’ has been removed to maintain the only one under methods section.

**Comment:** Please review your reference list to ensure that it is complete and correct. If you have cited papers that have been retracted, please include the rationale for doing so in the manuscript text, or remove these references and replace them with relevant current references. Any changes to the reference list should be mentioned in the rebuttal letter that accompanies your revised manuscript. If you need to cite a retracted article, indicate the article’s retracted status in the References list and also include a citation and full reference for the retraction notice.

**Response:** The reference list is complete and appropriately formatted.

**Responses to Reviewer 1 comments**

**Comment**: Kindly add a note about limitations of the study. For example, Limitations of the Patient Health Questionnaire in Identifying Anxiety and Depression. Ref Eack SM, Greeno CG, Lee BJ. Limitations of the Patient Health Questionnaire in Identifying Anxiety and Depression: Many Cases Are Undetected. Res Soc Work Pract. 2006 Nov 1;16(6):625-631. doi: 10.1177/1049731506291582. PMID: 24465121; PMCID: PMC3899353.

**Response**: Thanks for the suggestion. This limitation has been added to the ‘Limitations and future research’ section on page 23 lines 499–502 and states that…

*“Third, the PHQ-9 that was used to screen for depression has been reported to occasionally report false negative cases among the general population [94]. However, it has shown good psychometric properties in screening for depression among medical students in Uganda [33, 46, 58, 62, 65].”*

**Responses to Reviewer 3 comments**

**Comment:** In the Introduction the literature related to problematic Internet use should be deepened. For example, the following are suggested:

- *Ballarotto, G., Marzilli, E., Cerniglia, L., Cimino, S., & Tambelli, R. (2021). How does psychological distress due to the COVID-19 pandemic impact on internet addiction and Instagram addiction in emerging adults?. International journal of environmental research and public health, 18(21), 11382.*
- *Spada, M. M. (2014). An overview of problematic Internet use. Addictive behaviors, 39(1), 3-6.*
- *Tambelli, R., Cimino, S., Marzilli, E., Ballarotto, G., & Cerniglia, L. (2021). Late Adolescents’ Attachment to Parents and Peers and Psychological Distress Resulting from COVID-19. A Study on the Mediation Role of Alexithymia. International Journal of Environmental Research and Public Health, 18(20), 10649.*
- *Deutrom, J., Katos, V., & Ali, R. (2022). Loneliness, life satisfaction, problematic internet use and security behaviours: re-examining the relationships when working from home during COVID-19. Behaviour & Information Technology, 41(14), 3161-3175.*
- *Ballarotto, G., Volpi, B., & Tambelli, R. (2021). Adolescent attachment to parents and peers and the use of Instagram: The mediation role of psychopathological risk. International journal of environmental research and public health, 18(8), 3965.*
- *Özaslan, A., Yıldırım, M., Güney, E., Güzel, H. Ş., & İşeri, E. (2022). Association between problematic internet use, quality of parent-adolescents relationship, conflicts, and mental health problems. International Journal of Mental Health and Addiction, 20(4), 2503-2519*

**Response:** Thank you for your comment and for providing additional literature related to problematic internet use. We have now included a more detailed discussion of the relevant literature in the revised ‘Introduction’ section of our manuscript. We hope that the revised version increases the clarity and readability of our paper. Thank you again for your valuable feedback.

**Comment:** The authors used the English version of the tools. How come?

**Response:** Thank you for your question. This is very simple. All our participants were medical students who are proficient in English as they conduct all their university academic activities in this language. Therefore, we used the original English version of the scales. This has now been clarified in the revised manuscript on page 7 lines 209 to 210;

*“All data were collected in English language given that all the medical students in the present study were proficient in English because all their medical training is conducted in this language.”*
